# TRAF3 deficiency promotes metabolic reprogramming in B cells

**DOI:** 10.1038/srep35349

**Published:** 2016-10-18

**Authors:** Nurbek Mambetsariev, Wai W. Lin, Alicia M. Wallis, Laura L. Stunz, Gail A. Bishop

**Affiliations:** 1 Dept. of Microbiology, The University of Iowa, 3-403 Bowen Science Building, 51 Newton Road, Iowa City, IA 52242, USA; 2 Medical Scientist Training Program, The University of Iowa, Carver College of Medicine, 2206 MERF, Iowa City, IA 52242-2600, USA; 3 Immunology Graduate Program, 357 Medical Research Center, Iowa City, IA 52242-1182, USA; 4 Holden Comprehensive Cancer Center, University of Iowa Hospitals and Clinics, 200 Hawkins Drive, Iowa City, IA 52242, USA; 5 University of Iowa and DVA Medical Center, 601 Highway 6 West, Iowa City, IA 52246, USA; 6 Internal Medicine, 200 Hawkins Drive, Iowa City, Iowa 52242, USA.

## Abstract

The adaptor protein TNF receptor-associated factor 3 (TRAF3) is a critical regulator of B lymphocyte survival. B cell-specific TRAF3 deficiency results in enhanced viability and is associated with development of lymphoma and multiple myeloma. We show that TRAF3 deficiency led to induction of two proteins important for glucose metabolism, Glut1 and Hexokinase 2 (HXK2). This was associated with increased glucose uptake. In the absence of TRAF3, anaerobic glycolysis and oxidative phosphorylation were increased in B cells without changes in mitochondrial mass or reactive oxygen species. Chemical inhibition of glucose metabolism or glucose deprivation substantially attenuated the enhanced survival of TRAF3-deficient B cells, with a decrease in the pro-survival protein Mcl-1. Changes in Glut1 and Mcl-1 levels, glucose uptake and B cell number in the absence of TRAF3 were all dependent upon NF-κB inducing kinase (NIK). These results indicate that TRAF3 deficiency suffices to metabolically reprogram B cells, a finding that improves our understanding of the role of TRAF3 as a tumor suppressor, and suggests potential therapeutic strategies.

TRAF3 is an adaptor protein with diverse context and cell-specific roles^1^. B cell-specific deletion of *Traf3* in mice (B-*Traf3*^−/−^) results in markedly enhanced B cell survival and substantially increases incidence of B cell lymphoma, implicating TRAF3 as a tumor suppressor^2^,^3^. Studies of human tumors identified *TRAF3* mutations in nearly 20% of multiple myelomas and more than 15% of diffuse large B cell lymphomas^4^,^5^.

B cell survival and activation are linked to metabolic reprogramming. Chronic exposure to the pro-survival cytokine BAFF metabolically primes B cells by increasing respiratory capacity, while stimulation through the B cell receptor (BCR) or TLR4 increases glucose metabolism^6^,^7^. IL-4- mediated enhancement of B cell survival is also dependent upon glycolysis^8^. *In vivo* B cell-specific deletion of Glut1, a glucose transporter induced by activation through the BCR or TLR4, substantially reduces B cell number and inhibits antibody production^6^. Glut1 expression is also necessary to maintain elevated glucose metabolism and to promote survival in B cell acute lymphoblastic leukemia and multiple myeloma^9^,^10^. HXK2 is an inducible kinase that promotes glucose metabolism and cell survival and has been suggested as a therapeutic target in cancer^11^. HXK2 is upregulated in lymphocytes upon activation or cytokine stimulation^12^,^13^.

Although TRAF3 deficiency in B cells dramatically alters survival, the metabolic changes associated with this phenotype have not been explored. In this study, we show that TRAF3 deficiency was sufficient to induce expression of Glut1 and HXK2 in B cells. This in turn led to an increase in glucose uptake. TRAF3 deficiency resulted in metabolic reprogramming, characterized by an increase in both oxidative phosphorylation and anaerobic glycolysis, without changes in mitochondrial mass or production of reactive oxygen species (ROS). Inhibition of glucose metabolism promoted death of TRAF3-deficient B cells. Glucose was required for long term survival of these B cells, as well as maintenance of the pro-survival protein Mcl-1. In the absence of NF-κB inducing kinase (NIK), Glut1 and Mcl-1 were decreased in TRAF3-deficient B cells with associated decrease in glucose uptake. B-*Traf3*^−/−^ mice that lacked NIK had substantially reduced B cell numbers. Our results show that the pre-malignant survival phenotype of TRAF3-deficient B cells is accompanied by an altered metabolic state. These findings have important implications for pathogenesis and treatment of B cell malignancies promoted by TRAF3 deficiency.

## Results

### TRAF3-mediated regulation of glucose uptake

**Figure 1:**
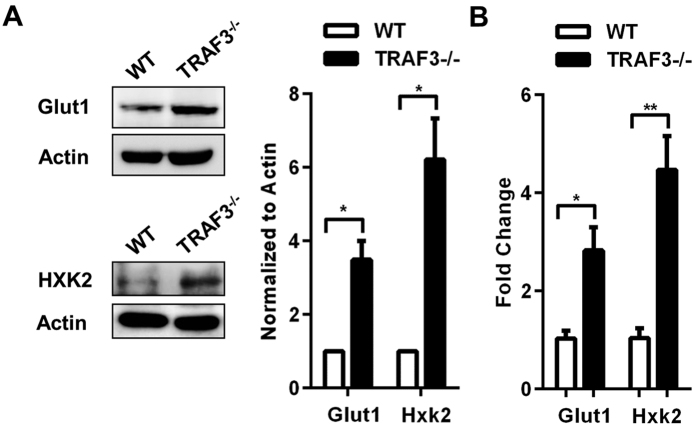
Induction of Glut1 and HXK2 in B cells in the absence of TRAF3. (**A,B**) B cells were isolated from littermate WT and B-*Traf3*^−/−^ mice. (**A**) Whole cell lysates were analyzed with Western blotting (WB) for Glut1 and HXK2 expression. Band intensities were quantified and normalized to actin. Graphs depict mean values ± SEM from three independent experiments. Full-length blots are presented in Supplementary Fig. S4. (**B**) *Glut1* and *Hxk2* mRNA levels were assayed with RT-PCR and analyzed as described in Materials and Methods. Data were normalized to GAPDH and fold change was determined using the comparative Ct method. N = 3 mice with mean values ± SEM shown. Student’s t test was used to evaluate differences for statistical significance in A and B (*p < 0.05, **p < 0.01).

Preliminary microarray data identified up-regulation of expression of genes encoding Glut1 and HXK2 in the absence of TRAF3, specifically in B cells. Glut1 is a glucose transporter and HXK2 is an inducible kinase that phosphorylates glucose, sequestering it intracellularly to be metabolized^14^. Glut1 and HXK2 are induced in B cells in response to antigen receptor stimulation, an important pro-survival signal^6^,^12^. B cells isolated from B-*Traf3*^−/−^ mice had increased protein abundance (Fig. 1A) and mRNA expression (Fig. 1B) of Glut1 and HXK2, compared to B cells isolated from wild type littermate controls (WT).

**Figure 2:**
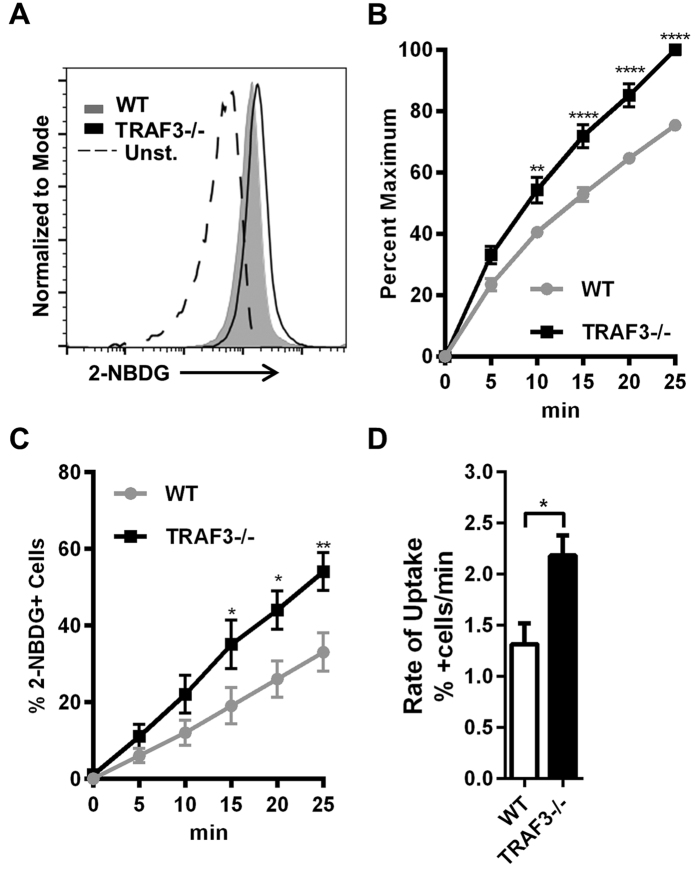
TRAF3 mediated regulation of glucose uptake in B cells. (**A–D**) B cells were isolated from littermate WT and B-*Traf3*^−/−^ mice. (**A–C**) Cells were incubated in the presence of 2-NBDG. Representative histogram of 2-NBDG uptake at 25 minutes, including an unstained sample, is shown (**A**). Median fluorescence intensities normalized as % maximum (**B**) and % of 2-NBDG positive cells (**C**) ±SEM at indicated time points are shown. Two-way ANOVA was used to analyze results for statistical significance and adjusted p value was calculated using the Bonferroni method (n = 3 independent experiments, *p < 0.05, **p < 0.01, ****p < 0.0001). (**D**) Rate at which cells take up 2-NBDG is shown, calculated from linear regression ± SEM from three independent experiments. Student’s t-test was used for statistical significance (*p < 0.05).

To investigate functional consequences of Glut1 and HXK2 induction, we used 2-(N-(7-Nitrobenz-2-oxa-1,3-diazol-4-yl)Amino)-2-Deoxyglucose (2-NBDG) as a fluorescent tracer of glucose uptake^15^. Measuring 2-NBDG uptake in B cells with flow cytometry revealed that loss of TRAF3 resulted in increased glucose transport (Fig. 2A,B). Consistent with increased Glut1 expression, TRAF3^−/−^ B cells also became 2-NBDG^+^ at a greater rate than did WT B cells (Fig. 2C,D). When imaged *in vivo* with positron emission tomography–computed tomography (PET-CT), older B-*Traf3*^−/−^ mice took up more glucose tracer, with a significant increase in the spleen compared to WT mice (Supplementary Fig. S1).

To determine if these changes were specific to B cells, we measured 2-NBDG uptake in T cells, sufficient or deficient in TRAF3. Mouse primary T cells deficient in TRAF3 showed no change in 2-NBDG uptake compared to WT littermate controls. Loss of TRAF3 did not lead to increase in Glut1 expression in mouse T cells and we were unable to detect any HXK2 expression. TRAF3 inhibition with shRNA in the HuT28.11 human T cell line did not alter 2-NBDG uptake or Glut1 and HXK2 expression (For T cell data see Supplementary Fig. S2). These findings indicate that loss of TRAF3 does not affect glucose metabolism in T cells. This is consistent with prior studies showing that unlike TRAF3-deficient B cells, T cells lacking TRAF3 do not have enhanced survival^16^.

### Metabolic consequences of B cell loss of TRAF3

**Figure 3:**
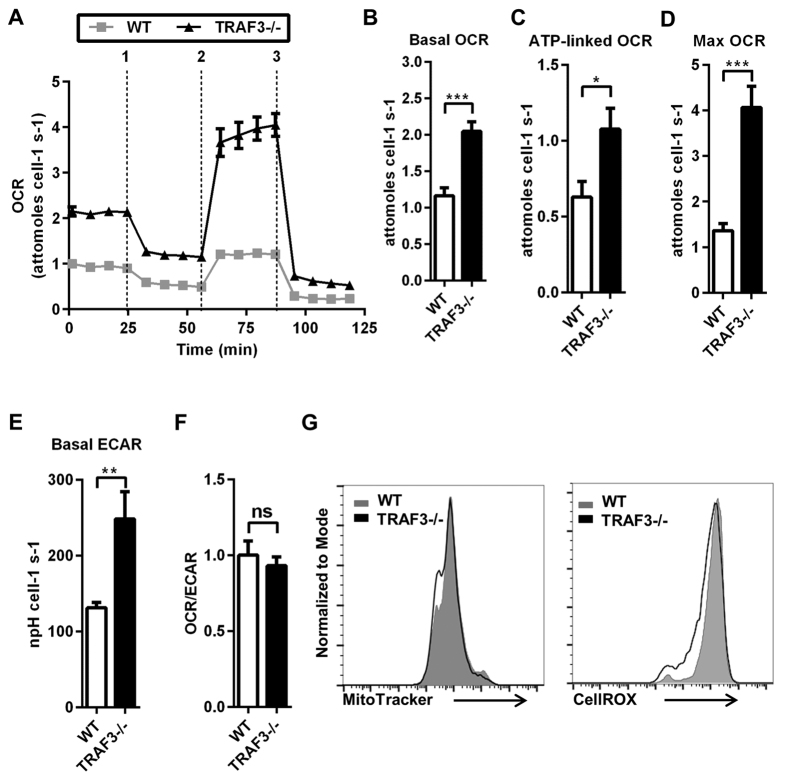
Metabolic consequences of B cell loss of TRAF3. (**A–G**) B cells were isolated from littermate WT and B-*Traf3*^−/−^ mice. (**A**) Representative plot of OCR over time with addition of oligomycin (1), FCCP (2) and antimycin ± rotenone (3). (**B**) Basal OCR was measured before the addition of oligomycin. (**C**) ATP-linked OCR was determined as the difference in OCR before and after addition of oligomycin. (**D**) Maximum OCR was measured after the addition of FCCP. (**E**) Basal ECAR was measured before addition of oligomycin. (**F**) OCR/ECAR is the ratio of basal OCR to basal ECAR. Data are from >4 independent experiments = SEM. Student’s t-test was used for statistical significance (ns = not significant, *p < 0.05, **p < 0.01, ***p < 0.001). (**G**) B cells were stained with MitoTracker Green or CellROX to measure mitochondrial mass and cellular ROS. Data are representative of two independent experiments.

Once transported into the cell, glucose can be metabolized via anaerobic glycolysis and oxidative phosphorylation^17^. To investigate if the increased glucose uptake of TRAF3-deficient B cells changes the metabolic status of the B cell, an extracellular flux analyzer, which measures oxygen consumption rate (OCR) and extracellular acidification rate (ECAR), was used to assay glucose utilization^18^. OCR is reflective of mitochondrial respiration and ECAR represents anaerobic glycolysis. TRAF3^−/−^ B cells had a substantially altered OCR profile compared to WT controls (Fig. 3A). Loss of TRAF3 led to increased basal, ATP-linked and maximum OCR (Fig. 3B–D). Interestingly, there was also an increase in basal ECAR (Fig. 3E). The balance between oxidative phosphorylation and anaerobic glycolysis can be expressed as the ratio of OCR/ECAR, which was unchanged in TRAF3^−/−^ B cells (Fig. 3F). This suggests that both anaerobic glycolysis and mitochondrial respiration were increased by loss of TRAF3 in B cells, analogous to the increase resulting from B cell activation^6^. These metabolic changes were not accompanied by an increase in mitochondrial mass or intracellular oxidative stress (Fig. 3G). Thus, loss of TRAF3 is sufficient to alter the metabolic profile of B cells.

### Role of glucose in survival of TRAF3^−/−^ B cells

**Figure 4:**
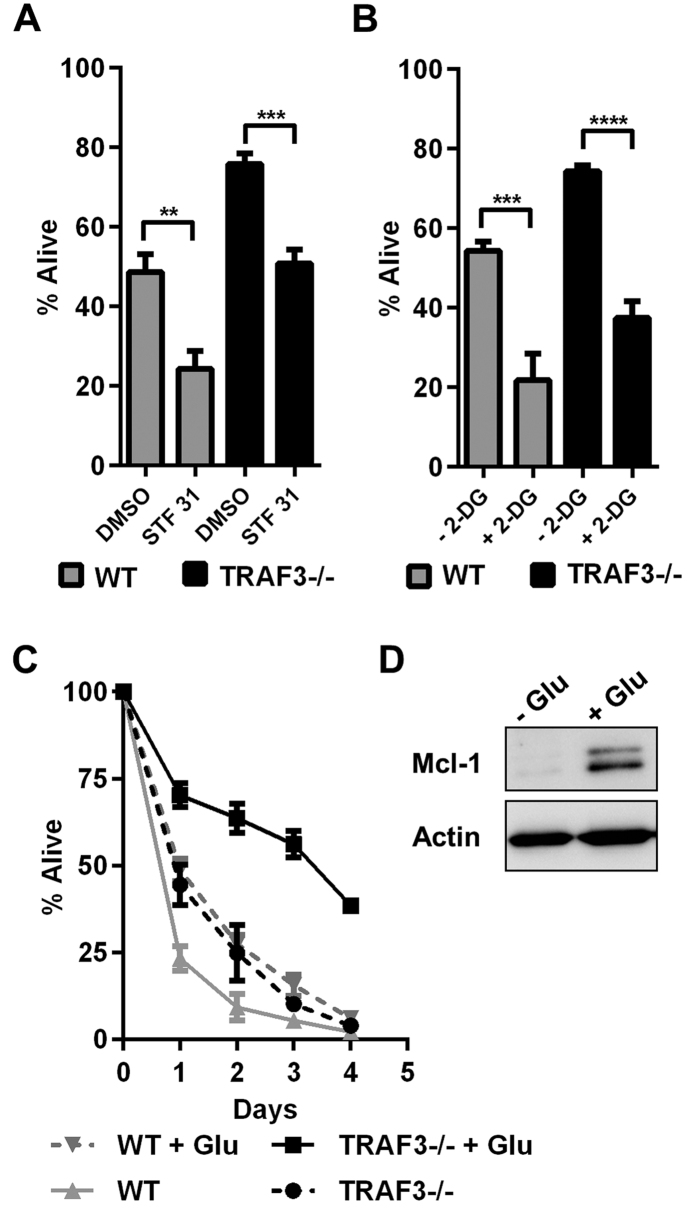
Role of glucose in survival of TRAF3^−/−^ B cells. (**A–D**) B cells were isolated from littermate WT and B-*Traf3*^−/−^ mice. (**A,B**) Cells were treated with 1 μM STF-31 (**A**) and 5 mM 2-DG (**B**) for 24 hours and viability was determined using PI exclusion. One-way ANOVA was used to analyze results for statistical significance and adjusted p value was calculated using the Bonferroni method (n = 6 independent experiments, **p < 0.01, ***p < 0.001, ****p < 0.0001). (**C**) Cells in medium containing glutamine, 1 mM pyruvate and 0.1% BSA were incubated in the presence and absence of glucose (Glu). Viability was determined via PI exclusion at indicated time points. Data are from four independent experiments = SEM. (**D**) Whole cell lysates were analyzed with WB for Mcl-1 protein expression after 48 hours in the presence and absence of glucose. Full-length blots are presented in Supplementary Fig. S4. Data are representative of three independent experiments.

TRAF3^−/−^ B cells display a dramatic increase in viability^2^ that is not seen in other TRAF3-deficient cell types^16^,^19^,^20^. Previous reports have implicated glucose as a nutrient important for cell survival^21^. We hypothesized that the survival phenotype resulting from B cell TRAF3 deficiency was dependent on glucose metabolism. STF-31 is a chemical inhibitor of Glut1 that is effective in targeting survival of malignant cells *in vivo* and *in vitro*^22^. Treatment of WT and TRAF3^−/−^ B cells with STF-31 resulted in attenuation of their survival (Fig. 4A) confirming the importance of Glut1 in B cell homeostasis. Similarly, treatment with the competitive glycolysis inhibitor, 2-deoxyglucose (2-DG), lowered B cell viability (Fig. 4B). Chemical inhibitors carry an intrinsic risk of non-specific effects. To test the contribution of glucose specifically to B cell survival, we cultured WT and TRAF3-deficient B cells in serum-free medium supplemented with pyruvate and glutamine as carbon sources, in the absence of glucose. This led to substantial cell death (Fig. 4C). WT B cells benefitted from glucose supplementation early in the course of the incubation, but after two days in culture the pro-survival glucose effect was almost completely diminished in these cells (Fig. 4C). In contrast, TRAF3^−/−^ B cells showed robust and sustained improvement in their survival in the presence of glucose, suggesting that long-term aberrant survival of B cells lacking TRAF3 is dependent upon glucose availability (Fig. 4C). Mechanistically, glucose promotes cell survival by increasing abundance of the Mcl-1 protein, an anti-apoptotic member of the Bcl-2 family^23^,^24^. We have recently shown that TRAF3^−/−^ B cells have elevated Mcl-1^25^. Glucose deprivation of TRAF3^−/−^ B cells resulted in reduction in Mcl-1 (Fig. 4D) indicating that glucose is important for maintaining pro-survival programming caused by the absence of TRAF3.

To determine the relevance of these findings for TRAF3 deficiency in human B cells, we analyzed four human B cell lymphoma-derived cell lines (see Supplementary Fig. S3). BJAB had much higher TRAF3 expression compared to Ramos, Ramos.2G6 and OCI Ly7 cell lines. Consistent with our mouse data, cell lines lower in TRAF3 had higher expression of Glut1. TRAF3 low/Glut1 high-expressing cell lines were also more susceptible to inhibition of glucose metabolism with STF-31 and 2-DG. Finally, glucose deprivation did not affect survival of high TRAF3-expressing BJAB cells, while promoting cell death in low TRAF3-expressing cell lines. These results implicate changes in glucose metabolism as an important driver of the enhanced survival and propensity to malignant transformation of B cells deficient in TRAF3.

### Role of NIK in TRAF3-mediated changes in glucose uptake and survival in B cells

**Figure 5:**
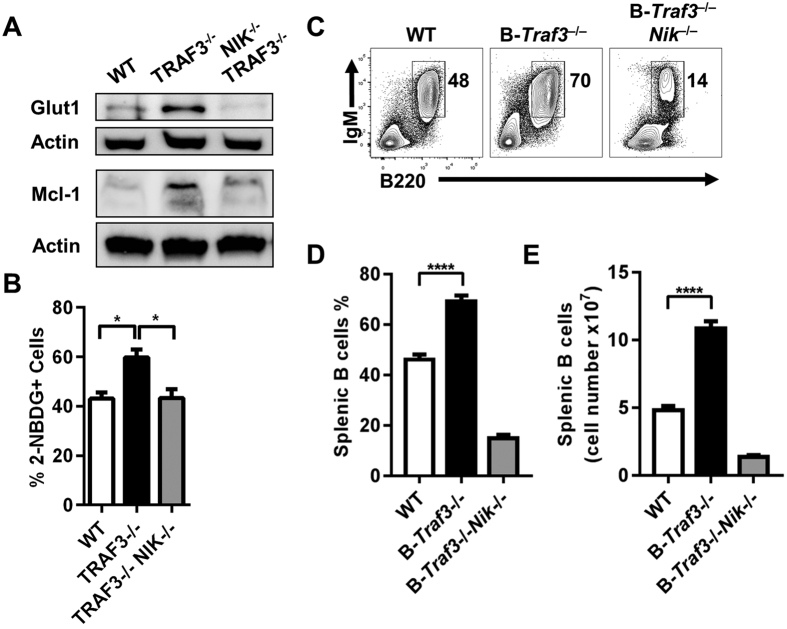
Role of NIK in TRAF3-mediated changes in glucose uptake and survival in B cells. (**A**) Whole cell lysates were analyzed with WB for Glut1 expression and Mcl-1. Full-length blots are presented in Supplementary Fig. S4. Data are representative of three independent experiments. (**B**) Cells were incubated with 2-NBDG for 30 minutes. One-way ANOVA was used to analyze results for statistical significance and adjusted p value was calculated using the Bonferroni method (n = 3 independent experiments, *p < 0.05). (**C**) Single-cell suspensions of splenocytes from the indicated mouse strains were stained with the indicated antibodies. Representative flow cytometry plots are shown. The outlined areas and the numbers indicate the frequencies of mature B cells (B cells: B220+IgM+). (**D**,**E**) Graphs represent mean ± SEM of the frequencies (**D**) and number (**E**) of mature B cells. One-way ANOVA was used to analyze results for statistical significance and adjusted p value was calculated using the Bonferroni method (n = 6 mice per group, ****p < 0.0001).

An important regulatory target of TRAF3 is NIK, a kinase that promotes activation of NF-κB, particularly non-canonical NF-κB2, and B cell homeostatic survival^26^. To test whether phenotypic changes in glucose uptake were dependent upon NIK, we crossed our B-*Traf3*^−/−^ mice onto a *Map14k*^−/−^ mouse (referred to as *NIK*^−/−^) to generate double- knockout B cells (NIK^−/−^TRAF3^−/−^). Loss of NIK in TRAF3^−/−^ B cells resulted in a decrease in both Glut1 and Mcl-1 expression (Fig. 5A), and in 2-NBDG uptake (Fig. 5B). *In vivo*, double-knockout mice had substantially reduced percentage and number of B cells in the spleen compared to both WT and B-*Traf3*^−/−^ mice (Fig. 5C–E). These findings highlight the importance of NIK in the altered metabolic phenotype and enhanced survival in B cells resulting from the absence of TRAF3.

## Discussion

Cancer pathogenesis has long been linked to metabolic changes required to maintain aberrant survival and growth of malignant cells, including increased utilization of glucose^21^. Cellular transformation and oncogenesis are intimately linked to altered metabolism that drives cancer progression^27^. TRAF3-deficient B cells gain a substantial survival advantage over WT B cells, potentially allowing them to acquire additional mutations with time. In a mouse model, this leads ultimately to development of lymphoma^3^. These findings are consistent with the occurrence of loss-of-function *TRAF3* mutations in human B cell malignancies^4^,^5^.

The metabolic impact of loss of TRAF3, however, has not been previously investigated. This study shows that B cells lacking TRAF3 undergo metabolic reprogramming, characterized by increased glucose uptake and utilization. Additionally, glucose availability is an important factor in their enhanced long-term survival. This suggests that in B cells, enhanced glucose metabolism occurs early in oncogenesis and precedes establishment of frank malignancy. These changes are similar to metabolic B cell responses to specific receptor stimulation, further highlighting the phenotypic similarities between lymphocyte activation and carcinogenesis^28^.

Targeting glucose metabolism has been suggested as a potential therapeutic strategy for cancer^29^. Inhibition of glucose utilization may also be useful in eradicating cells with pre-malignant alterations, such as TRAF3-deficient B cells, to prevent lymphomagenesis. The STF-31 inhibitor of Glut1 attenuated survival of WT and TRAF3^−/−^ B cells *in vitro* (Fig. 4) and B cell-specific Glut1 deletion substantially reduced B cell numbers *in vivo*^6^. Glucose deprivation also had an impact on WT and TRAF3^−/−^ B cells. These findings suggest that glucose metabolism is important for normal B cell homeostasis. However, extended survival of TRAF3-deficient B cells is inhibited in the absence of glucose, indicating that their premalignant phenotype is dependent upon glucose availability. This is in notable contrast to WT cells that succumb to apoptosis with prolonged culturing even in the presence of glucose. We predict that targeting glucose metabolism will affect both normal and malignant B cells, as do several successful currently approved strategies targeting B cell tumors, such as CD20-specific mAbs^30^, but will have a preferential impact on aberrant survival of TRAF3-deficient B cells. Our future plans include investigating the effect of *in vivo* deletion of the Glut1 transporter in TRAF3-deficient B cells on their survival and oncogenic potential. Glut1 also mediates intracellular transport of oxidized vitamin C, making tumor cells more susceptible to death induced by high doses of this compound^31^. The efficacy of therapeutic-dose vitamin C treatment in B cell malignancies in the context of TRAF3 deficiency is not yet known.

The established paradigm is that TRAF3 inhibits B cell survival by promoting degradation of NIK kinase, which in turn leads to inhibition of non-canonical NF-κB2 activation^32^. B cell-specific deletion of NIK leads to decreased mature B cell survival *in vivo* and *in vitro* and makes B cells unresponsive to BAFF stimulation^26^,^33^. Our findings show that increases in Glut1, Mcl-1 and glucose uptake in the absence of TRAF3 are dependent on NIK availability. Loss of NIK substantially reduces mature B cell numbers in B-*Traf3*^−/−^ mice even below the WT level. Our results support the concept model that enhanced survival of TRAF3-deficient B cells requires NIK, although our previously-published studies show that TRAF3 also regulates NIK-independent B cell survival pathways^25^.

This study focused on metabolic changes in B cells, potentially contributing to tumorigenesis. Clinical evidence also shows that obesity increases the long-term risk of lymphoma and multiple myeloma^34^,^35^. The role of systemic metabolic fitness in pathogenesis of hematological malignancies is poorly understood. The role of B cell TRAF3 deficiency in the context of obesity is an intriguing avenue of future research.

Our study focuses on the role of TRAF3 in B cell metabolism, but there is substantial evidence for its importance in maintaining systemic glucose homeostasis. Global TRAF3 deletion results in early post-natal lethality that is associated with hypoglycemia^36^. More recent work showed that hepatocyte-specific TRAF3 deletion protects mice from insulin resistance and hepatic steatosis^37^,^38^. Interestingly, analogous results were found with myeloid-specific TRAF3 deletion^39^. B cells have also been shown to promote inflammation in obesity-induced Type 2 diabetes^40^. An intriguing avenue of future research is the potential role of B cell specific TRAF3 deletion in systemic glucose metabolism.

The role of metabolic changes in T cells driving autoimmune disease has been aggressively explored^41^. B cells are also an important contributor to autoimmunity and a proven therapeutic target^42^. In addition to their B cell survival phenotype, B-*Traf3*^−/−^ mice also show a substantial increase in autoreactive antibodies^2^. The role(s) of changes in B cell metabolism in driving pathogenesis of autoimmune disease are unknown.

Metabolic reprogramming described here serves as an additional mechanism by which loss of TRAF3 promotes B cell survival and malignancy. Further elucidating the role of cell metabolism in B cell biology and cancer may provide new directions in treating B cell-driven diseases.

## Methods

### Mice

*Traf3*^flox/flox^ mice bred with *Cd19*-Cre (B-*Traf3*^−/−^) or *CD4*-Cre (T-*Traf3*^−/−^) mice and extensively backcrossed onto the C57BL/6 background were previously described^2^,^16^. For both strains, ‘WT’ refers to littermate controls. *Map14k*^−/−^ (also known as *Nik*^−/−^) mice were originally generated by Dr. Robert Schreiber (Washington University, St. Louis, MO) and provided by Dr. David Parker (Oregon Health Science University, Portland, OR). Mice of 2–4 months of age were used for all experiments except PET-CT imaging (see Supplement). All mice were maintained under specific pathogen-free conditions and were used in accordance with National Institute of Health guidelines under an animal protocol approved by the Animal Care and Use Committee at the University of Iowa. Similar numbers of male and female mice were used interchangeably in these studies.

### Mouse primary B cell isolation and culture

Splenic B cells were isolated by negative selection as previously reported^2^. Briefly, splenic B cells were isolated by anti-CD43 Ab-mediated negative selection, using a magnetic bead kit (Miltenyi Biotec, Auburn, CA) or mouse B cell isolation kit (STEMCELL, Vancouver, Canada), according to manufacturers’ protocols. Splenic T cells were isolated using a T cell isolation kit (STEMCELL), following the manufacturer’s protocol. Cells, including cell lines, were maintained in RPMI 1640 medium (Life Technologies, Grand Island, NY) containing 10 μM 2-β-mercaptoethanol (Sigma), 10% heat-inactivated FCS (Atlanta Biologicals, Atlanta, GA, USA), 2 mM l-Glutamine (Life Technologies), and 100 U/ml of penicillin-streptomycin antibiotics (Life Technologies). In glucose deprivation assays, cells were washed in sterile phosphate buffered saline (PBS, Life Technologies) to remove residual glucose-containing medium and incubated in glucose free RPMI 1640 medium supplemented by glutamine and 1 mM sodium pyruvate (Life Technologies) and 0.1% bovine serum albumin (BSA).

### Antibodies and reagents

Anti-Glut1 rabbit mAb (ab652) was purchased from Abcam (Cambridge, MA) and anti-HXK2 rabbit mAb (C64G5) from Cell Signaling (Danvers, MA). 2-NBDG, MitoTracker and CellROX dyes were purchased from Life Technologies. STF-31 was purchased from Tocris (Bristol, UK); 2-DG and propidium iodide from Sigma.

### 2-NBDG uptake

1 × 10^6^ cells per well were plated in a 96 well plate. Cells were incubated in 200 μL of B cell medium with a 2-NBDG concentration of 50 μM (25 μM for T cells) for indicated times. For mouse primary T cells, isolated T cells were stained with anti-CD8 mAb (53-6.7, BioLegend, San Diego, CA) and gated on CD8^+^ cells. After three washes with cold sterile PBS, samples were assayed with an Accuri C6 Flow Cytometer (BD Bioscience) and results were analyzed with FlowJo software (TreeStar, Ashland, OR).

### Extracellular flux analysis

Extracellular acidification rate and oxygen consumption rate were measured using a XF96 extracellular flux analyzer (Seahorse Bioscience, North Billerica, MA) at the Electron Spin Resonance facility at the University of Iowa. Cells were plated on poly-D-lysine-coated wells (Sigma) at a concentration of 6.7 × 10^5^ cells per well. OCR and ECAR measurements were normalized to cell number. Cells were plated in XF Seahorse medium with supplemental glucose and glutamine. The following inhibitor concentrations were used for the mitochondrial stress test: oligomycin, 1 μM; rotenone, 1.5 μM; antimycin A 1.5 μM; electron transport chain accelerator p-trifluoromethoxy carbonyl cyanide phenyl hydrazine (FCCP), 1.5 μM.

### Real-time PCR

RNA from mouse primary B cells was extracted with an RNeasy lipid extraction kit (Qiagen, Gaithersburg, MD) and cDNA synthesized using SuperScript III polymerase (Invitrogen). RT-PCR was performed on an ABI PRISM 7900 Sequence Detection System (Applied Biosystems, Grand Island, NY). Primers were purchased from Applied Biosystems (Mm00441480_m1 for Glut1 and Mm00443385_m1 for HXK2). Expression data were analyzed using the comparative Ct method^43^ and normalized to GAPDH expression.

### Flow cytometry analysis of splenic B cells

Single-cell suspensions were prepared from mouse spleen (SP) and red blood cells were lysed with ACK buffer (0.15 M NH_4_Cl, 10 mM KHCO_3_, 0.1 mM EDTA). For flow cytometric analysis, nonspecific staining was blocked with mAb to mouse CD16-CD32 (clone 93) and cells were stained with fluorescence-labeled mAbs specific for B220 (RA3-6B2) and IgM (II/41) (eBioscience, San Diego, CA), Flow cytometric analyses were performed using a BD FACS Verse at The University of Iowa Flow Cytometry Facility. Results were analyzed with FlowJo software.

## Additional Information

**How to cite this article**: Mambetsariev, N. *et al*. TRAF3 deficiency promotes metabolic reprogramming in B cells. *Sci. Rep.* **6**, 35349; doi: 10.1038/srep35349 (2016).

## Supplementary Material

Supplementary Information
