# Supplementary material for: TRAF3 deficiency promotes metabolic reprogramming in B cells
Source: Sci Rep. 2016 Oct 18;6:35349. doi: 10.1038/srep35349 (PMC5082756; doi:10.1038/srep35349)
Supplement: Supplementary Information [file srep35349-s1.pdf]

TRAF3 deficiency promotes metabolic reprogramming in B cells

Nurbek Mambetsariev[1,3,4], Wai W. Lin[1,3+], Alicia M. Wallis [1,3], Laura L. Stunz[1,5], and Gail A. Bishop[*].[1,2,3,5,6]

Dept. of Microbiology[1], Internal Medicine[2], Immunology Graduate Program[3], Medical Scientist Training Program[4] and Holden Comprehensive Cancer Center[5], University of Iowa and DVA Medical Center[6], Iowa City, IA

***Corresponding author:**

Gail Bishop

2296 CBRB, The University of Iowa

Iowa City, IA 52242

Phone: 319-335-7945

FAX: 319-335-9006

email: gail-bishop@uiowa.edu

[+]Present address: Sanford-Burnham-Prebys Medical Discovery Institute, La Jolla, CA

Supplemental experimental procedures

Integrated FDG-PET and CT scanning and image analysis

Imaging was done at the University of Iowa Small Animal Imaging Core as previously described[1]. 8 to 10 month-old mice with no signs of malignancy were anesthetized, administered 18F-FDG (7.59 +- 0.44 MBq) via the lateral tail vein and after a 60 minute uptake period placed prone in a heated (36°C) multimodality chamber (M2M Imaging, Cleveland, OH) in the PET scanner's gantry. PET list mode data were acquired for 15 min, using an Inveon small-animal PET/CT/SPECT imaging system (Preclinical Solutions, Siemens Healthcare Molecular Imaging, Knoxville, TN). In the same workflow, a CT image was acquired for attenuation correction purposes. Images were reconstructed using a 3D OP-MAP algorithm. Images were analyzed using PMOD v3.2 software (PMOD Technologies, Zurich, Switzerland) to quantify mean and maximum standardized uptake value (SUV).

Viral RNA transduction and constructs

A subclone of the human $CD4^+$ T cell line HuT transfected to stably express human CD28 (HuT28.11) was the generous gift of Dr. Arthur Weiss, University of California, San Francisco[2]. Cells were transduced to stably express the small hairpin (sh) RNA constructs shLUC (vector control) and shTRAF3 as previously described[3,4]. The following sequences were used for production of the shTRAF3 cell line (TRAF3-8 sense 5'GAACCTACCGGTCCGTGTGTCCCTGCTCATAAAGTAGTGAAGCCACAG 3' TRAF3-8 anti-sense 5' GTTCCGAATTCAAAAAATCGTGTGTCCCTGCTCATAAAGTACATCTGTGGCTTC3'; TRAF3-14 sense ,

5’GAACCTACCGGTAACTGGTTATCACTTGTGATAGTAGTGAAGCCACAG 3’

TRAF3-14 anti-sense

5’GTTCCGAATTCAAAAAACACTGGTTATCACTTGTGATAGTACATCTGTGGCTTC 3’). Both shTRAF3-8 and shTRAF3-14 were used together to produce the most effective inhibition of TRAF3 expression.

Supplementary references

1 Duncan, K. *et al.* (18)F-FDG-PET/CT imaging in an IL-6- and MYC-driven mouse model of human multiple myeloma affords objective evaluation of plasma cell tumor progression and therapeutic response to the proteasome inhibitor ixazomib. *Blood Cancer J* **3**, e165, doi:10.1038/bcj.2013.61 (2013).

2 Stein, P. H., Fraser, J. D. & Weiss, A. The cytoplasmic domain of CD28 is both necessary and sufficient for costimulation of IL-2 secretion and association with phosphatidylinositol 3'-kinase. *Mol Cell Biol* **14**, 3392-3402 (1994).

3 Bilal, M. Y., Vacaflores, A. & Houtman, J. C. Optimization of methods for the genetic modification of human T cells. *Immunol Cell Biol* **93**, 896-908, doi:10.1038/icb.2015.59 (2015).

4 Bilal, M. Y. & Houtman, J. C. GRB2 nucleates TCR-mediated LAT clusters that control PLC-γ1 activation and cytokine Production. *Front immunol* **6**, 141, doi:10.3389/fimmu.2015.00141 (2015).

Supplementary Figures

Fig. S1. *In vivo* glucose uptake by WT and B-*Traf3*$^{-/-}$ mice. Mice were imaged as described in supplementary methods. **A.** A representative FDG PET image of mice is shown. **B.** SUV Max and Average SUV in the spleen were quantified. Values for individual mice from two independent experiments and the mean values of each group are shown. Mann-Whitney test was used to determine statistical significance (*p<0.05).

Fig. S2. Role of TRAF3 in glucose uptake in T cells. A-B: T cells were isolated from littermate WT and T-*Traf3*$^{-/-}$ mice. **C-D:** HuT28.11 cells were stably transduced with vectors expressing shLuc (vector control) or shTRAF3. Cells were incubated for 15 minutes in the presence of 2-NBDG or were unstained and representative histogram is shown (A, C). Whole cell lysates were assayed for protein expression with Western blotting (B, D). Data are representative of three independent experiments.

Fig. S3. TRAF3 expression and susceptibility to inhibitors of glucose metabolism in B lymphoma cells. A. Whole cell lysates were analyzed with Western blotting for protein expression with representative blots shown. **B-D:** Cells were treated with 10μM STF-31 (B) and 10mM 2-DG (C) for 24 hours. Cells in medium containing glutamine, 1mM pyruvate and 0.1% BSA were incubated in the presence and absence of glucose (Glu) for 24 hours (D). Cell death was determined using PI exclusion. Data are from three independent experiments ± SEM (ns = not significant, **p<0.01, ***p<0.001).

Fig. S4. Full-size blots. A, B. Blots stained with anti-Glut1 and ant-Hxk2 antibodies are included in Fig. 1A. **C.** Blot stained with anti-Mcl-1 antibody included in Fig. 4D. **D,E.** Blots stained with anti-Glut1 and Mcl-1 antibodies are included in Fig. 5A. In our experience, Glut1 runs between 40-60 kDa, with some variability due to glycosylation.

Figure S1

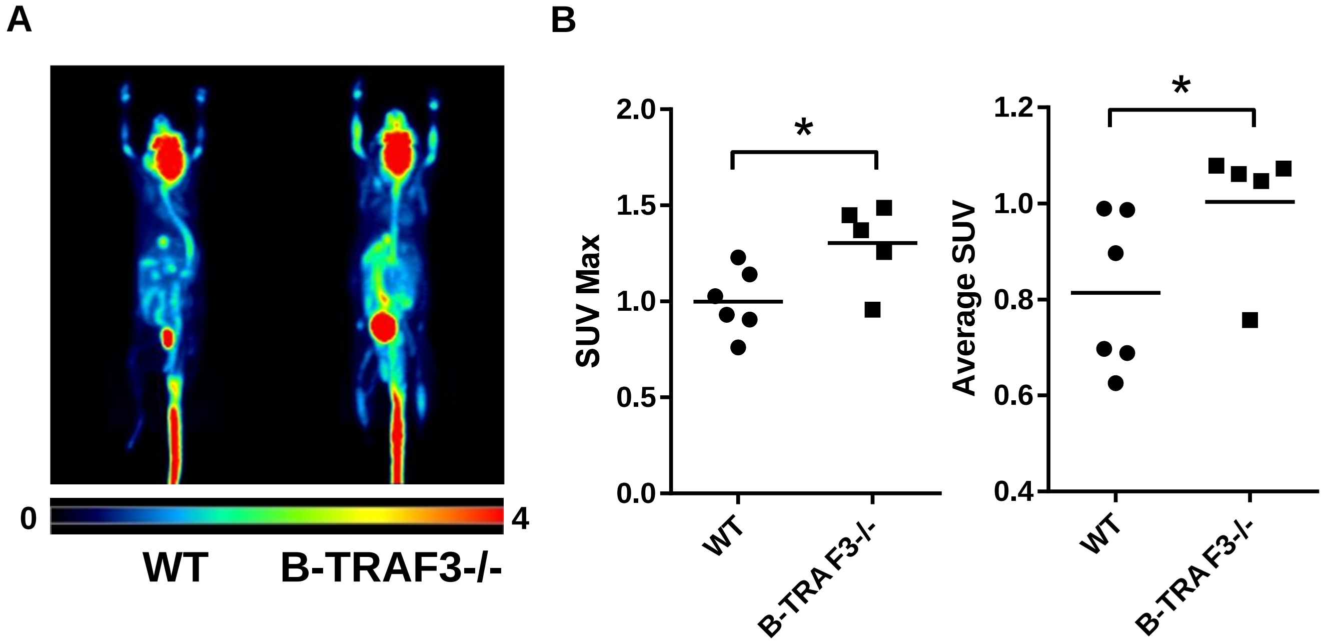
A
0
4
WT
B-TRAF3-/-
B
2.0
1.5
1.0
0.5
0.0
SUV Max
*
WT
B-TRA F3-/-
1.2
1.0
0.8
0.6
0.4
Average SUV
*
WT
B-TRA F3-/-

Figure S2

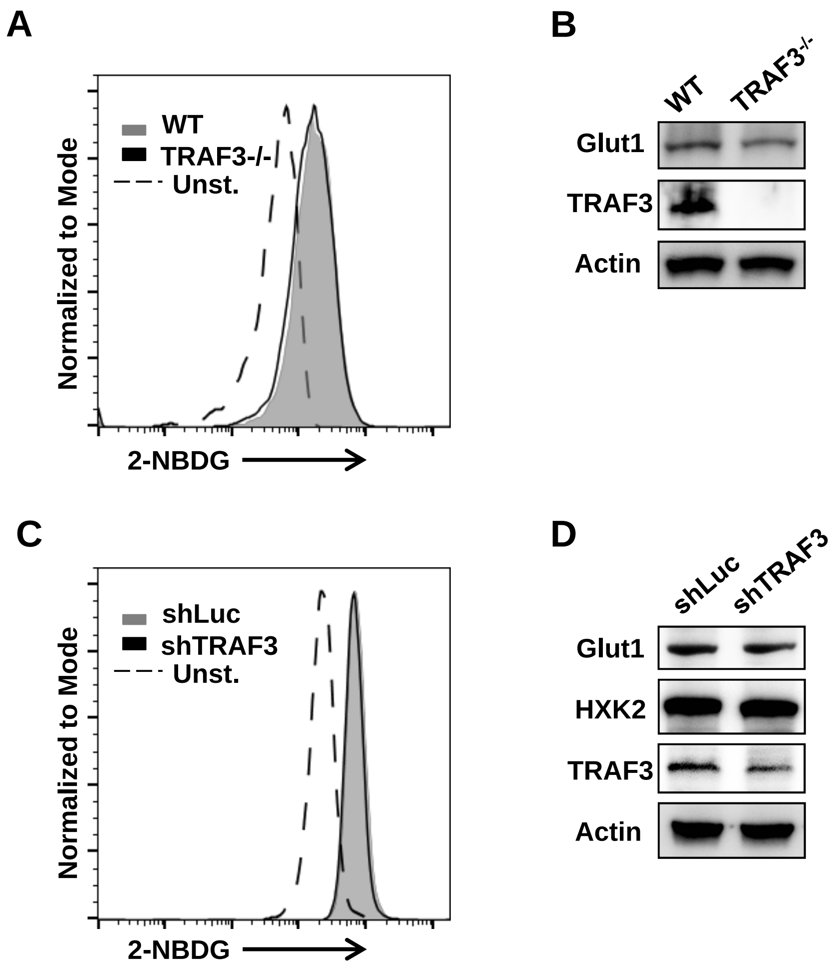
A
WT
TRAF3-/-
Unst.
Normalized to Mode
2-NBDG
B
WT
TRAF3-/-
Glut1
TRAF3
Actin
C
shLuc
shTRAF3
Unst.
Normalized to Mode
2-NBDG
D
shLuc
shTRAF3
Glut1
HXK2
TRAF3
Actin

Figure S3

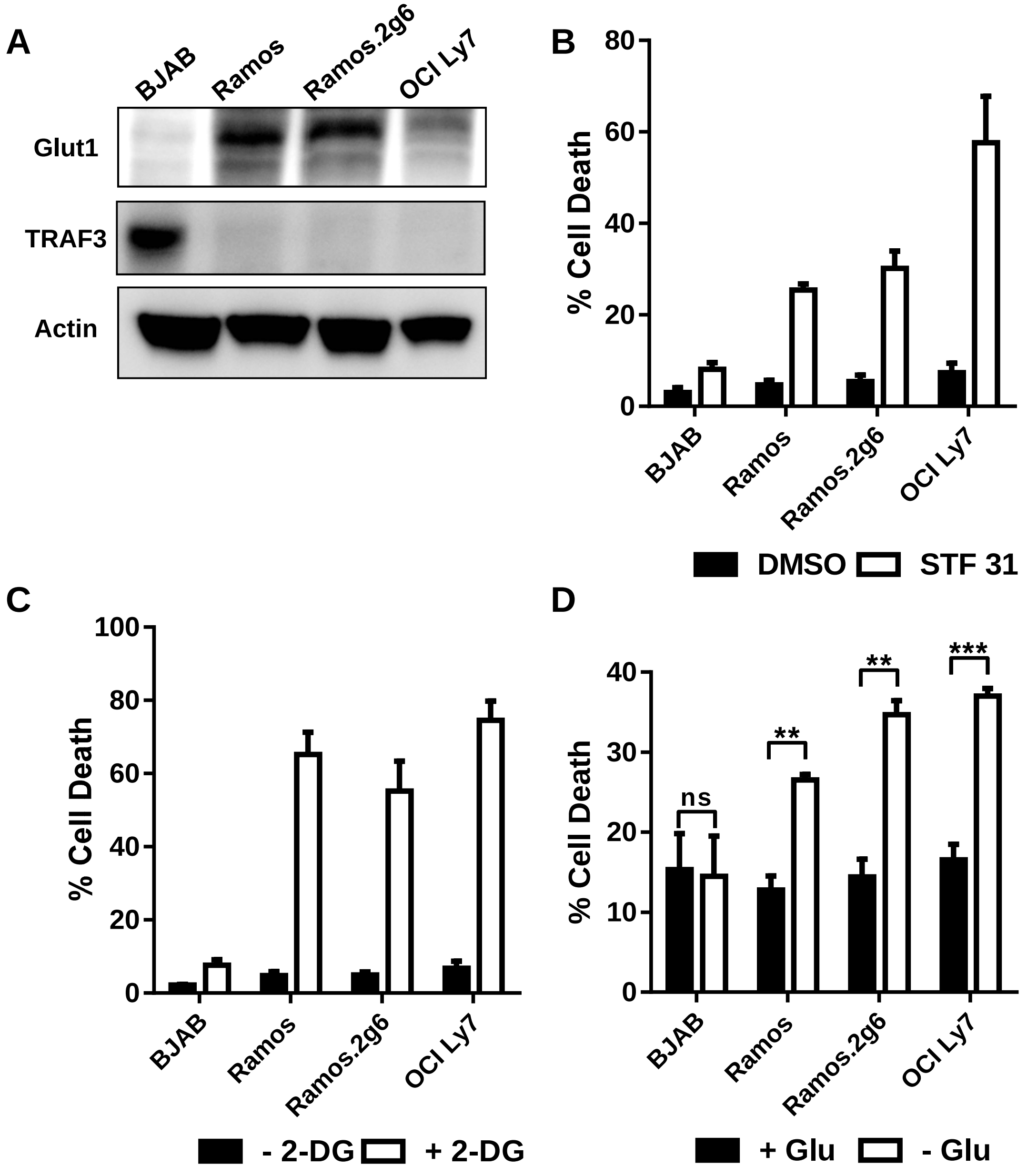

A
BJAB
Ramos
Ramos.2g6
OCI Ly7
Glut1
TRAF3
Actin
B
80
60
40
20
0
% Cell Death
BJAB
Ramos
Ramos.2g6
OCI Ly7
DMSO
STF 31
C
100
80
60
40
20
0
% Cell Death
BJAB
Ramos
Ramos.2g6
OCI Ly7
- 2-DG
+ 2-DG
D
40
30
20
10
0
% Cell Death
ns
**
**
***
BJAB
Ramos
Ramos.2g6
OCI Ly7
+ Glu
- Glu

Figure S4

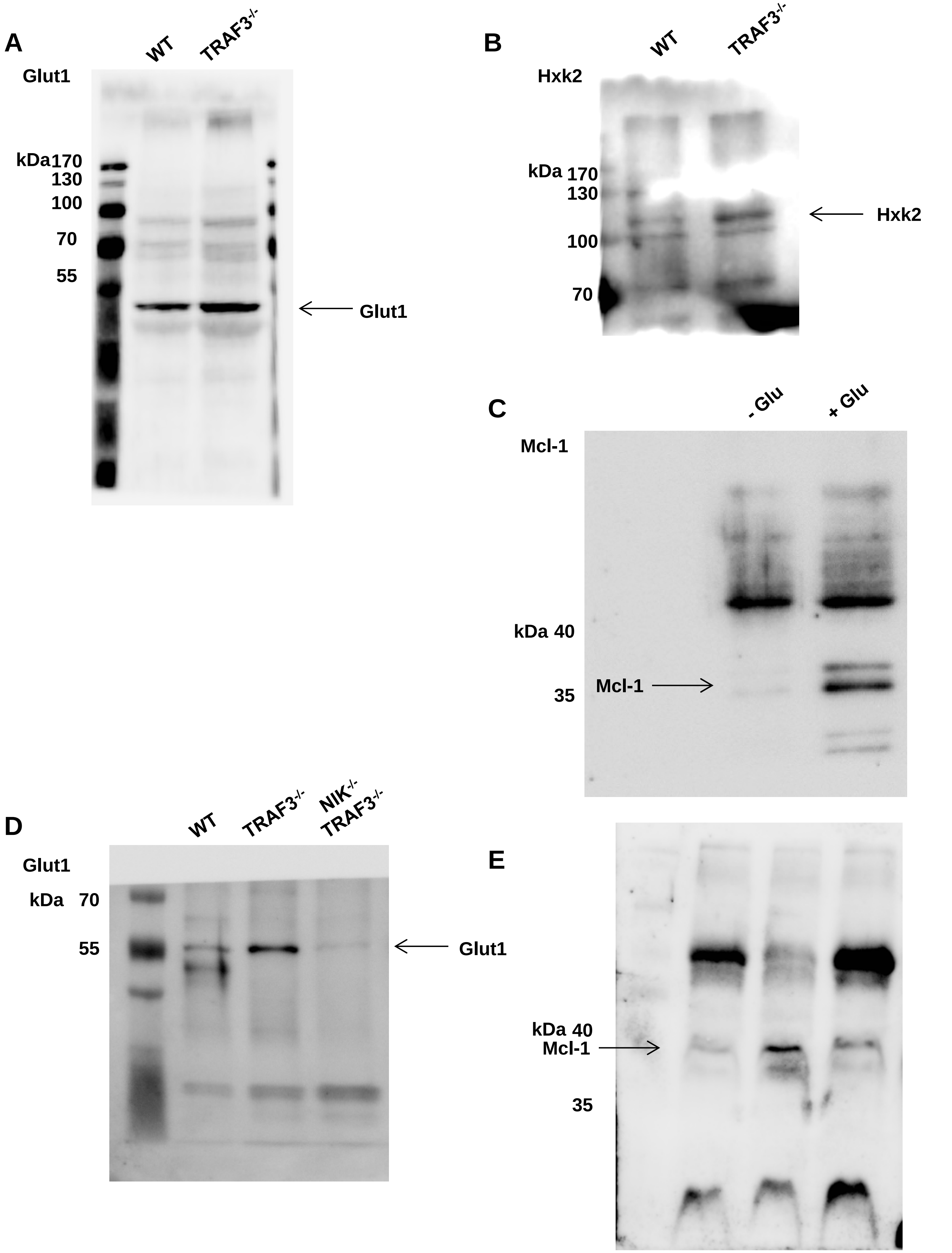

A
WT
TRAF3-/-
Glut1
kDa 170
130
100
70
55
Glut1
B
WT
TRAF3-/-
Hxk2
kDa 170
130
100
70
Hxk2
C
- Glu
+ Glu
Mcl-1
kDa 40
35
Mcl-1
D
WT
TRAF3-/-
NIK-/-
TRAF3-/-
Glut1
kDa 70
55
Glut1
E
kDa 40
Mcl-1
35
